
JOURNAL INFORMATION
==============================
NLM Title Abbreviation: ScientificWorldJournal
Journal ID: ScientificWorldJournal
NLM Journal ID: 101131163
Journal ID: TSWJ

The Scientific World Journal

ISSN: 2356-6140
EISSN: 1537-744X
Publisher: Wiley

ARTICLE INFORMATION
==============================
PMCID: PMC6009535
PMID: 29973805
DOI: 10.1100/tsw.2002.31
Article ID: 391676
Article version: 1
Subjects: Short Report

Secondary Structure Changes in a Calcium Binding Protein: Centrin

Pastrana-Rios Belinda *
Medina Zuleika
Ocana Wanda
Department of Chemistry, University of Puerto Rico Mayagüez Campus, Mayagüez, Puerto Rico 00681-9019, USA
*Belinda Pastrana-Rios: belinda@hpcf.upr.edu
Publication date: 2002
Electronic publication date: 2002 May 16
Volume: 2
First page: 62
Last page: 64
Copyright: Copyright © 2002 Belinda Pastrana-Rios et al.
Copyright year: 2002
License: This is an open access article distributed under the Creative Commons Attribution License, which permits unrestricted use, distribution, and reproduction in any medium, provided the original work is properly cited.
License URL: https://creativecommons.org/licenses/by/3.0/

==============================
Advances in Gene Technology: The Genome and Beyond –
Structural Biology for Medicine (Proceedings of the 2002 Miami
*Nature Biotechnology* Winter Symposium)
*TheScientificWorld* 2002, 2(S2), 62–64
ISSN 1532-2246; DOI 10.1100/tsw.2002.31

### SECONDARY STRUCTURE CHANGES IN A CALCIUM BINDING PROTEIN: CENTRIN

Belinda Pastrana-Rios, Zuleika Medina, and Wanda Ocaña
Department of Chemistry, University of Puerto Rico Mayagüez Campus, Mayagüez, Puerto Rico 00681-9019
Belinda@hpcf.upr.edu

**INTRODUCTION.** Centrin is a low-molecular-weight (20-kD), protein that belongs to the EF-hand superfamily of calcium-binding proteins. Local and overall changes were investigated for interactions between cations and phosphorylated and unphosphorylated *Chlamydomonas* centrin using Fourier transform infrared (FT-IR) spectroscopy. FT-IR spectral features studied included Amide I' band and the side-chain absorbance for aspartate the residues located almost exclusively at the calcium-binding sites in the spectral region of 1700–1500 $cm^{-1}$. The Amide I' band is exquisitely sensitive to changes in protein secondary structure and is often used to monitor changes in protein conformation. These spectral bands are complex and were further studied using two-dimensional FT-IR (2D-FT-IR) correlation along with curve-fitting routines. Using these methods the secondary structure contributions were determined for holo- and apo-centrin in its unphosphorylated form and phosphorylated holo-centrin. Thermal dependence revealed reversible melting with transition temperatures for each form of the protein also the phosphorylated holo-protein did not change significantly the secondary structure of the protein.

**METHOD**. *Chlamydomonas* centrin was overexpressed off a pt7-5 plasmid construct in *E. coli* BL21 $\lambda DE_3$ induced with IPTG (Gibco BRL, Ann Arbor, MI) to yield milligram quantities of the desired protein. The bacterial cells were lysed and the resulting supernatant was subjected to affinity column chromatography and strong anion exchange column chromatography. The purified sample was analyzed by SDS-PAGE, Western Blot, TOF-MS, and partial amino acid sequencing revealing the protein to be >99% pure.

To generate the phosphorylated protein, the sample was incubated at 30$^{o}$C with protein kinase A (PKA), DTT, and ATP overnight. Phosphorylated and the unphosphorylated samples were then extensively dialyzed against the appropriate buffer for each experiment: (1) FT-IR experiments of holo-centrin or phosphorylated holo-centrin would require 50 mM Hepes buffer containing 4 mM $CaCl_2$, 4 mM $MgCl_2$, and 150 mM NaCl at pH 7.4; and (2) FT-IR experiments of apo-centrin 50 mM Hepes buffer containing 4 mM EGTA, 4 mM EDTA, and 150 mM at pH 7.4 (3) for the titration experiments the dialyzed apo-centrin was subjected to a second dialysis with 50 mM Hepes buffer containing 2 mM EGTA, 2 mM EDTA, and 150 mM at pH 7.4.

Spectra were collected following complete H→D exchange in the centrin preparation. The sample was deposited between two 25 mm $CaF_2$ or $AgCl_2$, windows with a 40-μm spacer. A reference cell was prepared similarly. The instrument was an FTS-40 Bio-Rad (Cambridge, MA) or a Mattson Infinity Series with custom sample shuttle and interface. Typically, 512 scans were coadded, apodized with a triangular function, and Fourier transformed to provide a resolution of 4 $cm^{-1}$ with data encoded every 2 $cm^{-1}$. For the thermotropic studies, spectra were collected at sequential increments of temperature, allowing for equilibrium, and used in the 2D-FT-IR correlation analysis.

2D-FT-IR correlation analysis was performed using MathCad 2000 (MathSoft, Inc., Cambridge, MA) software. The curve-fitting routines were done using Grams (Galactic Industries Corp., Salem, NH) program. Origin 6 program was used to plot curve-fitted data.

**RESULTS.** The titration of apo-centrin as analyzed by 2D correlation analysis (Fig. 1) revealed the sequential secondary structure changes that occur during calcium binding and confirmed the apo- and holo-spectral features observed for this protein.

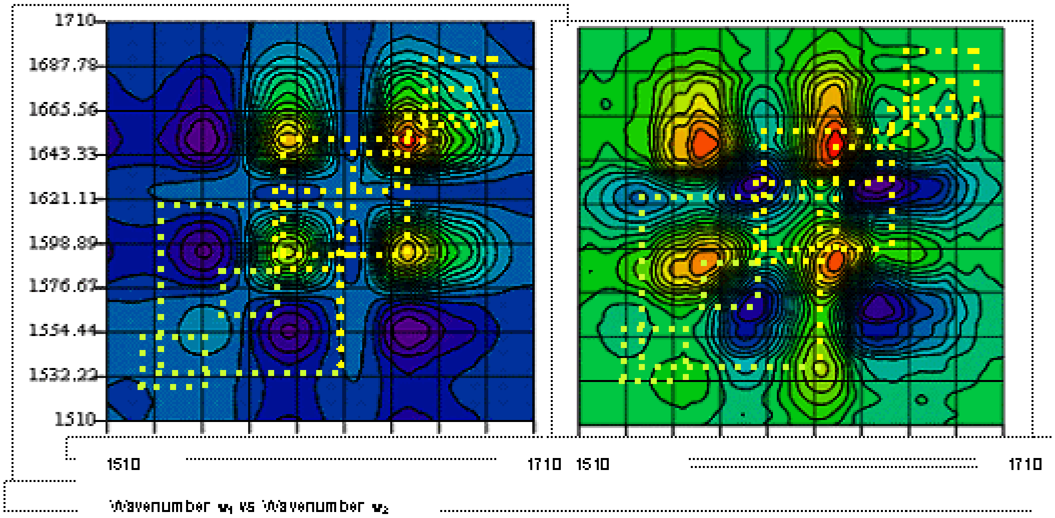

**FIGURE 1.** 2D correlational plots for the calcium titration experiment of Ccen; (a) synchronous and (b) asynchronous plot.

In addition, as shown in Fig. 2 the spectral features for apo- , holo-, and phosphorylated holocentrin.

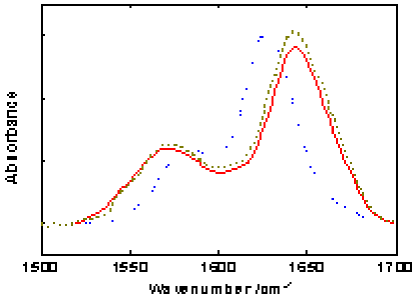

**FIGURE 2.** Superimposed spectra of the three forms of Ccen; (blue) apo-centrin, (red) holo-centrin, (gold) phosphorylated holo-centrin.

**DISCUSSION.** There are small changes in the spectral features of the Amide I' band when spectra obtained in the presence of cations yet the differences observed in the absence of cations are dramatic. Suggesting that there are secondary structural changes and different self-association states for this protein.

**REFERENCES**

1. Huang, B., Mengersen, A., and Lee, V.D. (1988) [pdb AC: P05434] J. Cell Biol. 107, 133–140.
2. Moncrief, N., Kretsinger, R., and Goodman, M. (1990) J. Mol. Evol. 30, 522–562.
3. Salisbury, J.L. and Floyd, G. (1978) Science 202, 975–977.
4. Weber, C., Lee, V.D., Chazin, W.J., and Huang, B. (1994) J. Biol. Chem. 269, 15795–15802.
5. Wiech, H., Geier, M.B., Paschke, T., Spang, A., Grein, K., Steinkotter, J., Melkonian, M., and Schiebel, E. (1996) J. Biol. Chem. 271, 22453–22461.
6. Lutz, W., Lingle, W.L., McCormick, D., Greenwood, T.M., and Salisbury, J.L. (2001) J. Biol. Chem. 276, 20774–20780.
